# GRM1 as a Candidate Gene for Buffalo Fertility: Insights from Genome-Wide Association Studies and Its Role in the FOXO Signaling Pathway

**DOI:** 10.3390/genes16020193

**Published:** 2025-02-04

**Authors:** Wangchang Li, Haiying Zheng, Duming Cao, Anqin Duan, Liqing Huang, Chao Feng, Chunyan Yang

**Affiliations:** 1 Guangxi Key Laboratory of Buffalo Genetics, Reproduction and Breeding, Guangxi Buffalo Research Institute, Chinese Academy of Agricultural Sciences, Nanning 530001, Chinaduanaq321@163.com (A.D.); 18269051851@163.com (L.H.);; 2 Guangxi Key Laboratory of Animal Breeding, Disease Control and Prevention, College of Animal Science & Technology, Guangxi University, Nanning 530004, China; liwangchang1019@163.com (W.L.);; 3 Key Laboratory of Buffalo Genetics, Breeding and Reproduction Technology, Ministry of Agriculture and Rural Affairs, Nanning 530001, China

**Keywords:** buffalo, production traits, genome-wide association study

## Abstract

Background: Water buffaloes represent a crucial genetic resource for the global dairy industry, yet enhancements in their production performance remain relatively constrained. The advent of advanced sequencing technologies, coupled with genome-wide association studies (GWASs), has significantly boosted the potential for breeding superior-quality water buffalo. Methods: An integrated genomic analysis was performed on sequencing data from 100 water buffaloes, utilizing the high-quality UOA_WB_1 genome assembly as a reference. This study particularly emphasized reproduction-related traits, with a focus on age at first calving (AFC). Results: Our analysis revealed two significant single-nucleotide polymorphisms (SNPs). Based on these genetic markers, the *GRM1* gene was identified as a candidate gene. This gene shows substantial involvement in various reproduction-associated pathways, including the FOXO signaling pathway, calcium signaling pathway, and estrogen signaling pathway. Conclusions: The identification of *GRM1* as a candidate gene provides a robust theoretical basis for molecular breeding strategies aimed at enhancing fertility in water buffaloes. These findings offer critical scientific support for optimizing breeding programs, thereby improving overall production efficiency.

## 1. Introduction

Water buffaloes play a crucial role in both meat and milk production, with their milk contributing significantly to the global dairy sector. Specifically, buffalo milk accounts for over 15% of global milk yield, highlighting the essential role of this species in the dairy industry. This statistic underscores the importance of focusing on buffalo welfare, productivity, and reproductive efficiency to ensure the continued growth and sustainability of this vital livestock sector [1]. Buffalo milk is distinguished by its elevated levels of fat, protein, and minerals when compared to cattle milk, which contributes to its nutritional superiority and economic value. This nutritional profile is a key factor in the greater prevalence of buffaloes in Asia, where they outnumber many other livestock species. The unique composition of buffalo milk not only enhances its suitability for dairy products but also reinforces the strategic importance of buffaloes in the agricultural economies of the region [2]. One critical aspect of enhancing buffalo milk production lies in improving reproductive efficiency, particularly through the optimization of age at first calving (AFC). AFC is a crucial reproductive trait that directly impacts the overall productivity and sustainability of buffalo herds. Identifying genetic markers and pathways that regulate AFC could lead to significant improvements in reproduction rates [3]. By pinpointing these genetic factors, we can develop strategies to shorten the AFC, thereby increasing the number of calves produced over the lifetime of each buffalo. This would not only enhance the reproductive efficiency of buffalo populations but also contribute to the overall economic viability of buffalo farming. Therefore, understanding and manipulating the genetic determinants of AFC is an essential area of focus for researchers, farmers, and stakeholders aiming to maximize the potential of the buffalo milk industry [4].

Traditional breeding methods for buffaloes face significant challenges due to various factors, including unstable estrus conditions and prolonged calving intervals. These issues complicate the breeding process and highlight the need for innovative approaches to enhance reproductive efficiency and success [5]. Modern genetic technologies offer promising strategies to address these challenges. Single-nucleotide polymorphisms (SNPs) represent one of the most fundamental and prevalent forms of genetic variation. Whole-genome sequencing is a powerful technique that allows for the identification of genetic variations across an organism’s entire genome. This method not only provides valuable insights into intrinsic genomic information but also plays a crucial role in the study of human diseases, crop improvement, and livestock breeding [6]. Whole-genome association analysis is a potent tool for investigating complex genetic traits and identifying candidate genes. By analyzing genetic variations and polymorphisms throughout the entire genome, this approach facilitates the detection of genomic regions and genes associated with specific traits of interest [7]. For instance, applying whole-genome association analysis to buffalo breeding can help identify SNPs linked to key reproductive traits, such as age at first calving (AFC) [8]. Understanding and leveraging these genetic markers can lead to more effective breeding programs, ultimately enhancing the reproductive performance and overall productivity of buffalo herds. Therefore, integrating advanced genetic technologies into buffalo breeding practices is essential for overcoming current limitations and maximizing the economic potential of buffalo farming [9].

Globally, in previous years, several studies have been conducted, analyzing the correlation between SNPs and reproductive traits. In their study, Islam et al. [10] embarked on a comprehensive genome-wide association analysis involving 167 buffaloes, with the primary objective of discovering novel candidate genes linked to traits such as body weight and production performance. This analysis aimed to shed light on the genetic underpinnings of these economically important characteristics. The study employed the 90K Axiom Buffalo SNP Array to detect SNPs within a range of 2000 base pairs upstream of the buffalo *FABP* gene [11]. They identified SNP sites within a specific region and performed an association analysis to examine the relationship between these SNPs and various reproduction-related traits. Despite the increasing interest in the application of whole genome association analysis in studying reproductive-related traits in water buffaloes, the number of studies conducted in this area remains limited when compared to the extensive body of research in cattle. This highlights the need for further investigation in this field to deepen our understanding of the genetic factors underlying reproductive traits in water buffaloes.

In this study, using four statistical models, linkage-disequilibrium iteratively nested keyway (Blink) [12], fixed and random model circulating probability unification (FarmCPU) [13], generalized linear models (GLM_Q) [14], and settlement of mixed linear models under progressively exclusive relationships (SUPER) [15,16], we fully identified two high-confidence SNPs. Through variant annotation and QTL mapping, we detected GRM1 candidate genes that could potentially affect the reproductive traits. By understanding these factors, we aim to provide valuable insights that will guide our future research and contribute to the development of more effective breeding strategies.

## 2. Materials and Methods

### 2.1. Ethics Statement

All finished work was conducted in accordance with national and international guidelines. The protocol for this study was approved by the Attitude of the Animal Care & Welfare Committee of the Guangxi Buffalo Research Institute (Approval Code: GXU2019-021).

### 2.2. Phenotypes and Animal Resources

This study utilized data collected from 100 water buffaloes at the Guangxi Buffalo Research Institute in China from 2000 to 2021. The sample comprised the following: 1 Guangxi local water buffalo (DB), 44 hybrid water buffaloes (ZB), 23 Nili-Ravi water buffaloes (NB), and 32 Murrah water buffaloes (MB). The Murrah water buffaloes are purebred animals originally imported from India, while the Nili-Ravi water buffaloes are purebred animals initially introduced from Pakistan. The hybrid water buffaloes included in this study were produced by crossing Nili-Ravi and Murrah breeds. The target trait of this study is age at first calving (AFC).

### 2.3. Sample Collection and Sequencing

Blood samples from water buffaloes were obtained through tail vein puncture by utilizing a vacuum blood collector. Genomic DNA was extracted from the blood using the phenol/chloroform method, and its integrity and yield were evaluated via agarose gel electrophoresis. The DNA libraries were sequenced on the Illumina sequencing platform (Illumina HiSeqTM 2000) by Genedenovo Biotechnology Co., Ltd. (Guangzhou, China).

### 2.4. Alignments and Variant Identification

The clean reads were aligned to the reference genome (UOA_WB_1) using BWA-MEM (v0.7.17) with default settings [10]. Then, Samtools (v1.9), Picard tools (v3.1.1), and GATK (v4.0) were used for SNP detection [17,18]. All detected SNPs underwent filtering through the “Variant Filtration” module of GATK, using the following standard parameters: variants with Quality Depth (QD) < 2; FS (Phred-scaled *p*-value using Fisher’s exact test for strand bias detection) > 60; MQRankSum (Z-score of the rank sum of the Phred-scaled mapping qualities) < −12.5; ReadPosRankSum (Z-score of the rank sum of the Phred-scaled position bias estimations) < −8; MQ (root mean square of the mapping quality) < 40.0; the mean sequencing depth of variants (across all individuals) was limited to less than 1/3× and more than 3×; SOR (strand odds ratio) > 3.0; the maximum missing rate was less than 0.1; and SNPs were limited to two alleles.

### 2.5. Variation Filtering

The presence of rare alleles (alleles with low frequency within the population), high rates of missing data, and substantial heterozygosity at specific loci can introduce anomalies in population analysis and whole-genome association studies. Therefore, we aligned the processed reads to the reference genome (UOA_WB_1). Subsequently, we employed PLINK (v1.9) software to filter the detected loci based on standard criteria [19]. The filtering process involved stringent adherence to several criteria: exclusion of non-biallelic SNPs, removal of those with a minor allele frequency below 0.05, discarding SNPs with a missing genotype rate exceeding 30%, and further limiting the analysis to SNPs with a heterozygosity ratio below the threshold of 0.8. This was all executed using robust PLINK (v1.9) software.

### 2.6. Principal Component Analysis

GCTA (v1.92.2) is a robust tool for the analysis of whole-genome complex traits [20]. In this study, we utilized GCTA (v1.92.2) and PLINK (v1.9) software to perform PCA (Principal Component Analysis) using the selected SNP markers. This analysis enabled us to derive the variance accounted for by each PC (principal component) and the score matrix representing the samples’ positions within each PC.

### 2.7. Population Structure Analysis

Population structure analysis offers valuable insights into the ancestry and composition of individuals, rendering it an exceptionally effective approach for elucidating genetic relationships. To validate the outcomes of the PCA, we performed a population structure analysis. Model-based population structure inference methods typically assume that the markers utilized for analysis are independent of one another. Consequently, prior to initiating the analysis, it is essential to execute marker independence filtering, which is based on the assessment of linkage disequilibrium between markers. In this analysis, we utilized PLINK (v1.9) and Admixture software (v1.3) [21] to perform marker filtering for population structure analysis. In our analysis, we implemented a 100 kb step size and a 10 nucleotide (nt) window size, and we removed one marker from each pair of markers with an r^2^ value greater than 0.2. Specifically, we removed the marker with the higher physical position from each pair of markers with a high degree of linkage disequilibrium. As a result of implementing the aforementioned filtering strategy, we retained a total of 272,462 markers for the population structure analysis. Utilizing the filtered SNP markers, we conducted a principal component analysis (PCA) using PLINK to investigate the population structure and clustering patterns. The PCA results were visualized to illustrate the relationships among the first three principal components, providing insights into the genetic diversity and relatedness within the population. Additionally, we employed Admixture software (v1.3) to perform an in-depth analysis of population structure, estimating the proportion of ancestry from K ancestral populations and identifying subpopulations within the dataset. In our analysis, we explored the cross-validation (CV) error for various k-values using Admixture software (v1.3), ranging from 2 to 9. We utilized PopHelper software (v2.2.7) [22] to generate bar plots illustrating the genetic composition of each sample within every subgroup. By systematically testing these k-value hypotheses, we aimed to identify the optimal number of clusters that would provide the most meaningful and informative partitioning of the studied populations.

### 2.8. Genome-Wide Association Mapping

A genetic relationship matrix was constructed based on the genotype using TASSEL (version 5.2.86) [23], and principal component analysis (PCA) was implemented with PLINK software (version 1.9.0). We used GAPIT (version 3.0) [16] to identify candidate SNPs associated with AFC traits, which is widely used to identify QTLs [24], and analyze the association between the molecular markers and traits of the mixed population based on 4 models: GLM (Q) [14], SUPER [15], FarmCPU [13], and Blink [12]. To correct the influence of population structure and reduce the false positive rate, the kinship matrix and the first 3 PCA scores were fitted as random and fixed effects, respectively [25]. Manhattan and quantile-quantile (Q-Q) plots were drawn with the CMplot package [26] in R software (version 4.2.2), and Venn diagrams were plotted using the ggvenn package (https://CRAN.R-project.org/package=ggvenn, accessed on 1 October 2024) in R software (version 4.2.2).

Generalized Linear Models are a widely employed and versatile statistical method for data analysis [14]. In the present study, we utilized Generalized Linear Models for conducting a genome-wide association analysis.

The model of GLM (Q) [14] is as follows:

y = Xα + Zβ + Wμ + e

where y is the phenotypic trait to be studied; α is the fixed effect, which is another factor affecting the phenotype and generally refers to population structure; β is the marker effect, which refers to SNPs; μ is the random effect, X, Z, and W are the incidence matrices for α, β, and μ, respectively; and e is the residual of the model.

The model of SUPER [15] is as follows:

y = Xα + Zβ + Wμ + e(1)

L(y|β, σa2, σe2, s, b)(2)

L(y|α, β, σa2, σe2, s^, b^)(3)

where Equation (1) has the same parameters as the GLM (Q) model, and Equation (2) is the solution of all unknown parameters under which Equation (1) determines the observations y with maximum likelihood, where σa2 and σe2 are the genetic variance and residual variance, s and b are the number and size of bins, and s^ and b^ are the estimates to maximize the number and size of bins, respectively.

The model of FarmCPU [13] is as follows:

yi = Si1 b1 + Si2 b2 + … + Sit bt + Hij dj + ej(4)

yi + ui + ei(5)

where yi is the observed value of the ith individual; Si1, Si2,…, Sit are the genotypes of the pseudo quantitative trait nucleotides (QTNs); b1, b2,…, bt are the corresponding effects of the pseudo QTNs; Hij is the jth genetic marker by the ith individual; dj is the effect value for the marker; and ei is the residuals of the model. yi and ui in Equation (5) are the same as in Equation (4), and ui is the total genetic effect of the ith individual.

The model of Blink [12] is as follows:

yi = Si1 b1 + Si2 b2 + … + Sit bt + Sij dj + ej(6)

yi = Si1 b1 + Si2 b2 + … + Sit bt + ei(7)

BIC = –2LL + 2tLn(n)(8)

where LL is the log likelihood, t is the number of pseudo QTNs, Ln is the natural log, n is the number of individuals, and the symbolic meanings of Equations (6) and (7) are the same as those of Equation (6) in FarmCPU.

The outcomes are presented through Manhattan plots and Q-Q plots. SNPs exhibiting *p*-values below the specified threshold of 0.05/N (number of SNP) are identified as highly significant SNPs. When a reference genome is available, candidate genes are determined by including those genes that are physically positioned within a 50 kb genomic region surrounding the significant SNPs. DbSNP [27] is a database specifically designed by the NCBI to store genetic variation information. We used dbSNP to determine whether the SNPs we identified are located in the coding regions of genes.

### 2.9. Pathway Enrichment and Protein–Protein Interaction

Genes often work in concert to perform specific biological functions. Pathway-based analysis is a valuable approach for understanding the roles of genes in these complex processes. The KEGG (Kyoto Encyclopedia of Genes and Genomes) database [28] stands as one of the foremost publicly accessible resources for pathway-related data. To identify significantly enriched metabolic and signal transduction pathways among CAGs (Candidate Associated Genes) relative to the entire genome context, pathway enrichment analysis was performed. The method for calculating enrichment is consistent with that employed in Gene Ontology (GO) [20] analysis:

$$P=1-\sum_{i=0}^{m-1}\frac{\left(\begin{array}{c} M\\ i \end{array})(\begin{array}{c} N-M\\ n-i \end{array}\right)}{\left(\begin{array}{c} N\\ n \end{array}\right)}$$

In this context, N signifies the total count of genes with KEGG annotations, while n denotes the number of CAGs within N. M represents the total number of genes annotated to particular pathways, and m is the number of CAGs in M. Following the calculation of the *p*-value, it was corrected using False Discovery Rate (FDR) adjustment, with an FDR value of 0.05 or less being set as the threshold. Pathways that met this criterion were categorized as significantly enriched pathways in CAGs. Finally, we utilized the String database to identify genes that are significantly represented in pathways and created a protein–protein interaction map. Gene Ontology (GO) and Kyoto Encyclopedia of Genes and Genomes (KEGG) analysis were performed using OmicShare tools, a free online platform for data analysis (http://www.omicshare.com/tools, accessed on 1 October 2024).

### 2.10. Statistical Analysis

Statistical analyses were performed using the SPSS 18.0 software package (SPSS Science, Chicago, IL, USA). Experimental data were subjected to *t*-test and ANOVA analyses, with a significance threshold set at *p* < 0.05. Graphs were generated using GraphPad Prism 8 software (GraphPad, Santiago, USA). Data are presented as the mean ± standard deviation (SD).

## 3. Results

### 3.1. Phenotypic Value Statistics of the Traits

**Figure 1:**
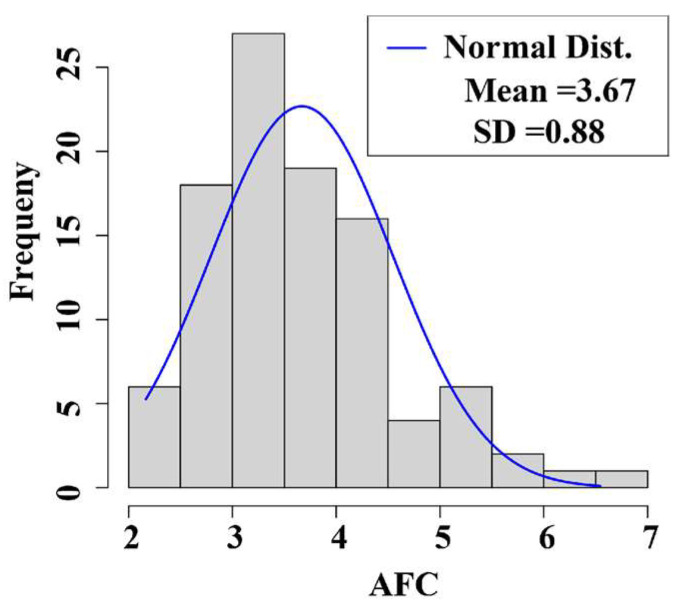
AFC histogram. AFC, age at first calving (year).

During the phenotypic evaluation of the buffaloes, we analyzed the age at first calving (AFC) as a key production trait. Specifically, for the AFC phenotypic value analysis: The mean value of AFC was recorded as 3.67 years; detailed phenotypic data statistics are presented in Figure 1 and Table 1. Our results, supported by relevant literature, clearly indicate that AFC is a critical factor influencing reproductive efficiency. Previous studies have identified 4 years as a significant threshold, suggesting that buffaloes with an AFC greater than 4 years tend to exhibit lower reproductive performance. This comprehensive analysis provides insights into the reproductive performance of buffaloes, with particular emphasis on the AFC trait.

In this study, a comprehensive analysis of genome-wide variations led to the identification of 2,208,174 genetic markers. Among the detected genetic markers, 2,012,270 were identified as SNPs and 195,904 were classified as insertion–deletion (Indel) variants. Following stringent filtering criteria, a refined set of 272,462 markers was retained, comprising 255,453 SNPs and 17,009 Indels.

### 3.2. Population Structure

**Figure 2:**
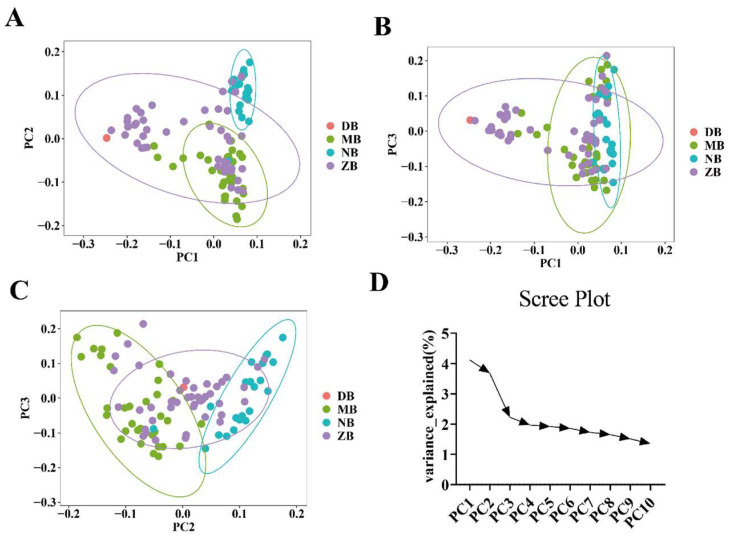
Graphs displaying the sample clustering obtained from PCA through three two-dimensional scatter plots, namely scatter (**A**), scatter (**B**), scatter (**C**), and scree plots (**D**). The percentage of variance explained by each PC is noted in parentheses. In the scatter plots, colored circles represent four different groups: DB, MB, NB, and ZB correspond to 1 DB, 32 MB, 23 NB, and 44 ZB, respectively.

Upon obtaining PCA scores, the samples under investigation can be visualized via a scatter plot that utilizes the values of the first three principal components as axes. Referring to Figure 2, in scatter plots Figure 2A, it is evident that the majority of individuals within herds MB and NB are distinctly isolated from one another. In scatter plot Figure 2B, we can observe clustering, particularly in groups MB and ZB, where these two clusters overlap and are closely grouped together in multiple instances. In scatter Figure 2A,C, it is evident that the herds are broadly segregated into three distinct clusters. One cluster predominantly consists of NB herds, whereas the other two clusters are primarily composed of MB, ZB, and DB herds, respectively.

**Figure 3:**
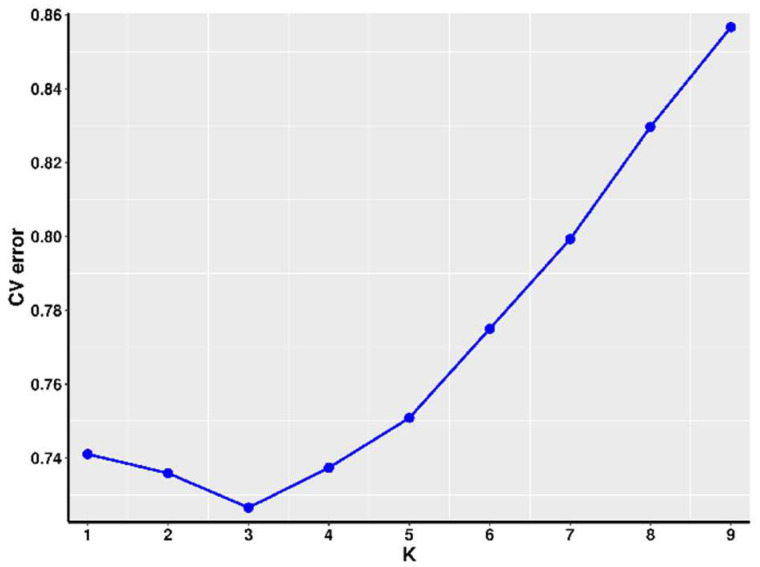
The line graph illustrating the cross-validation error rate is depicted, with the number of sample clusters delineated along the *x*-axis and the corresponding cross-validation error rate indicated on the *y*-axis.

To establish the optimal number of clusters (k), we used Admixture software and evaluated the cross-validation error rates. The Admixture algorithm performs model-based clustering and estimates the proportion of ancestry from K ancestral populations. By minimizing the cross-validation error rates, we identified the value of k = 3 that best fits our data. Figure 3 displays the line graph depicting the cross-validation error rate.

**Figure 4:**
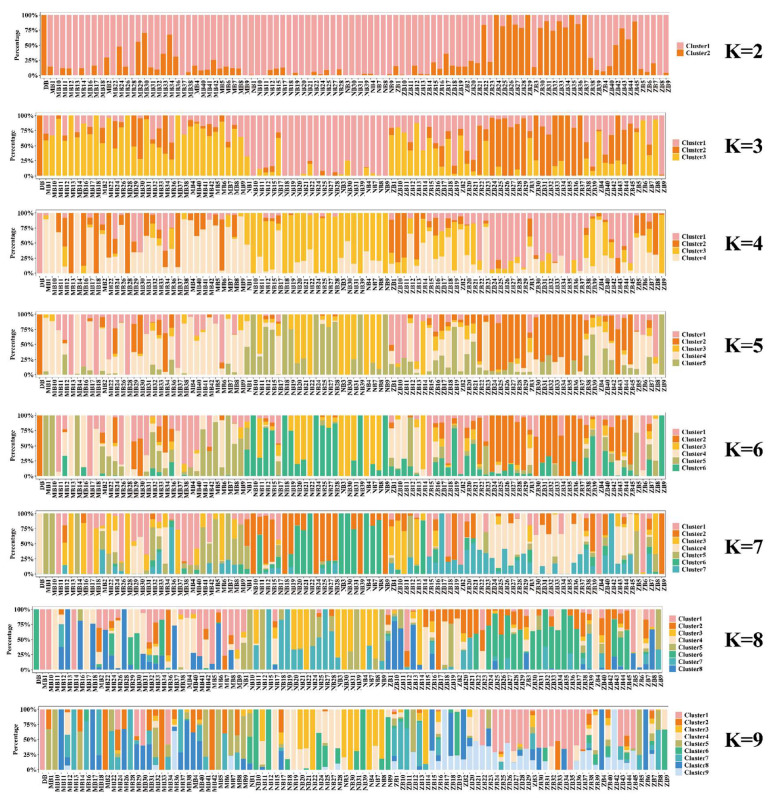
Genetic bar chart illustration for K-means clustering with varying numbers of clusters (K = 2 to 9).

To simulate the population classification and genetic ancestry of each sample across varying numbers of subgroups (K = 2–9), we utilized PopHelper software (v2.2.7) [22] to generate bar plots illustrating the genetic composition of each sample within every subgroup. The results are presented in Figure 4, where each color corresponds to a distinct cluster for each K-value. From the line graph of the cross-validation error rate, it is evident that the optimal number of clusters is K = 3. Similarly, as observed in the bar graph (Figure 3) depicting the genetic composition of the samples, when K = 3, it is the optimal number of clusters for these 100 buffaloes. This finding is consistent with the results obtained from the PCA analysis. Consequently, we conclude that these 100 buffaloes can be effectively divided into three distinct subgroups.

### 3.3. Results of the Genome-Wide Associations

**Figure 5:**
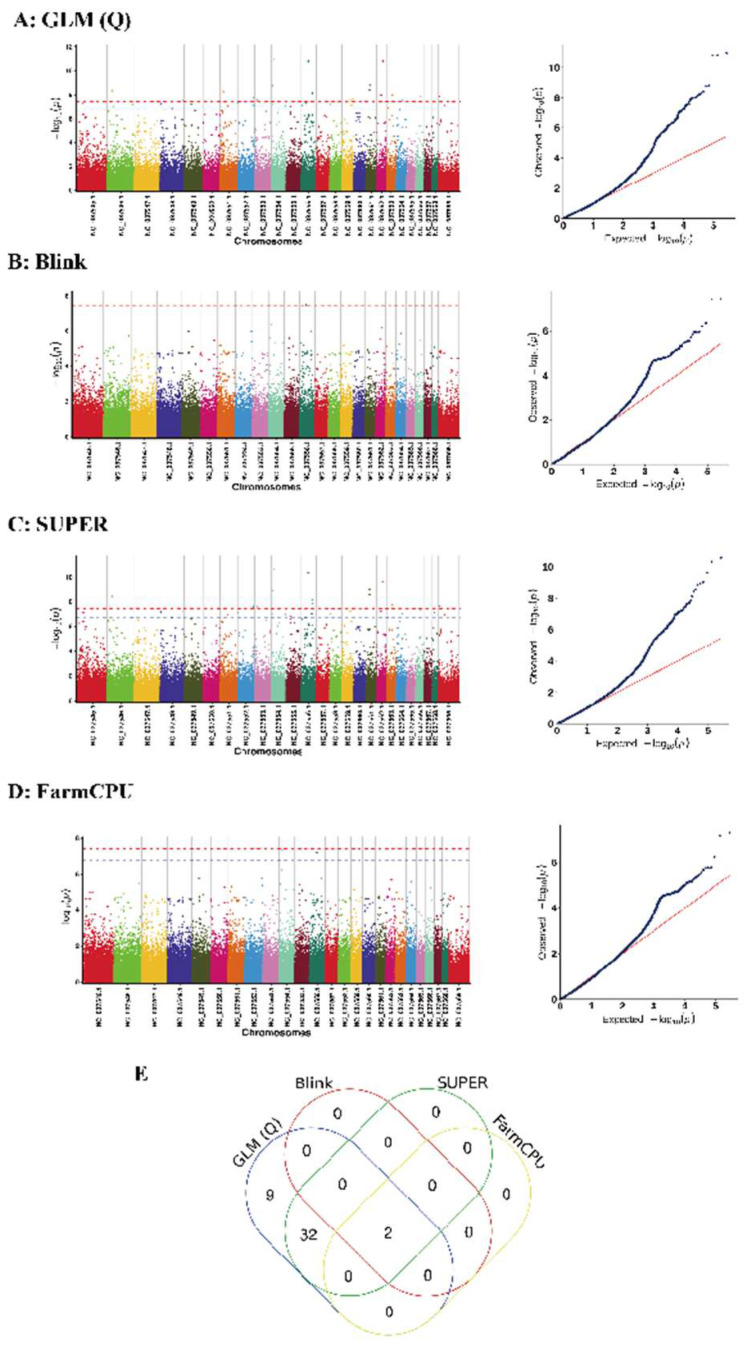
Association analysis of age at first calving (AFC) traits in water buffaloes using different GWAS methods. (**A**) GLM-Q, (**B**) BLINK, (**C**) SUPER, (**D**) and FarmCPU. (**E**) Venn plot of significant SNPs identified using all four methods. The Manhattan plot on the left, created using the qqman package, illustrates the *p*-values for SNP markers across 25 chromosomes (comprising 24 autosomes and one X chromosome). The blue line delineating the Manhattan plot signifies the significance threshold, determined by 0.05/N (number of SNPs). Markers that surpass this threshold are deemed significant. The plot on the right is a Q-Q plot, where the *x*-axis denotes the observed values of the markers, and the *y*-axis represents the expected values, which have been transformed into the −10 log scale.

Following the calculation of *p*-values for the SNP loci using a generalized linear model, we constructed a Manhattan plot and a Q-Q plot, as presented in Figure 5.

The leftmost plot is a Manhattan plot, where the visually discernible blue line, running parallel to the *x*-axis, serves as the critical demarcation line. In the Manhattan plot, the points that rise above the threshold line, which is the blue line paralleling the *x*-axis, signify significant loci. Upon identifying the significant loci that surpass the threshold line in the Manhattan plot, the subsequent step involves documenting the relevant information pertaining to these significant loci.

The plot on the right is the Q-Q plot, where the points in the bottom left corner fall along the line, suggesting that the observed *p*-values align closely with the expected values. The points exhibit a distinct upward deviation from the diagonal in the upper right corner, which signifies that the observed *p*-values exceed the anticipated values. The presence of these points, which denote significant loci, across all four plots underscores the validity of the analytical model, suggesting its appropriateness in capturing the underlying patterns. Following this, the genes situated within a 50 kb range of the significant loci are carefully selected and earmarked as candidate genes.

In our GWAS study, we analyzed significant loci using four different GWAS methods (GLM-Q, BLINK, SUPER, and FarmCPU) (Figure 5A–D). Our analysis revealed that SUPER and GLM(Q) identified 34 significant SNPs (Figure 5E), which were annotated to 30 genes (Detailed pathway information for these candidate genes: Supplementary Table S1). To ensure the robustness and reliability of our findings, we focused on the significant loci identified using all four methods (Blink, SUPER, GLM(Q), and FarmCPU). This stringent approach enabled us to pinpoint two statistically significant SNPs and identify one candidate gene (Table 2) within a 50 Kb range surrounding these loci, specifically associated with the trait age at first calving (AFC).

### 3.4. Kyoto Encyclopedia of Genes and Genomes Pathway Analysis of Candidate Genes

**Figure 6:**
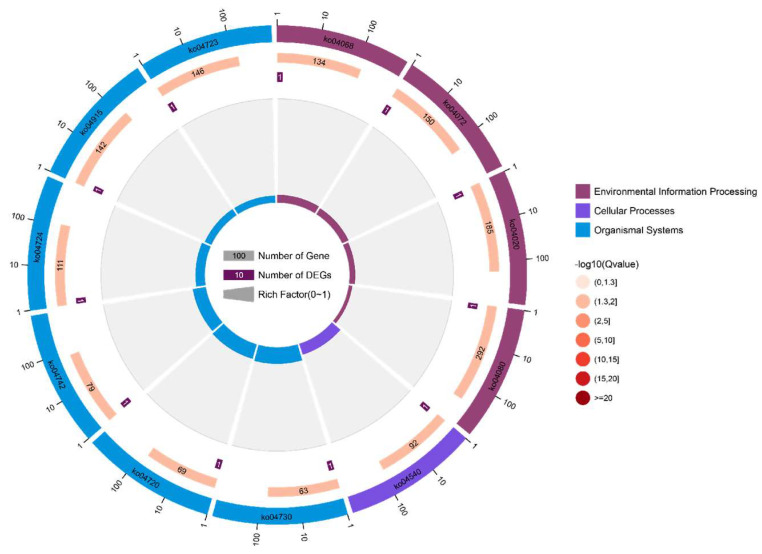
KEGG analysis of candidate genes. The enrichment circle diagram shows the KEGG analysis of the top 20 pathways. Four circles from the outside to the inside. First circle: The classification of enrichment; outside the circle is the scale of the number of genes. Different colors represent different categories. Second circle: The number and *p*-values of the classification in the background genes. The more genes, the longer the bars, the smaller the value, and the redder the color. Third circle: Bar chart of the total number of candidate genes. Fourth circle: rich factor value of each classification (the number of candidate genes in this classification divided by the number of background genes). Each cell of the background helper line represents 0.1, and the color coding signifies the statistical significance of the corresponding enrichment.

The functional enrichment cycle diagram displayed the top 20 KEGG pathways (Figure 6, Supplementary Table S2), classified by –log10 (*p*-value), which revealed that the candidate genes were mainly enriched in five KEGG_A_class pathways, including environmental information processing, cellular processes, and organismal systems. Among these pathways, environmental information processing included the FOXO signaling pathway (ko04068), the phospholipase D signaling pathway (ko04072), the calcium signaling pathway (ko04020), and neuroactive ligand–receptor interaction (ko04080); cellular processes included gap junction (ko04540). In conclusion, our data suggest that the identified candidate genes play a crucial role in regulating fertility performance, particularly in terms of age at first calving (AFC), by modulating the FOXO signaling pathway, phospholipase D signaling pathway, and calcium signaling pathway. These findings provide valuable insights into the molecular mechanisms underlying fertility and could inform future breeding strategies to enhance reproductive performance in water buffaloes.

### 3.5. Significant Association of Fertility with SNP Validation

Table 3 presents the results of individual genotyping for a single key locus in water buffaloes, NC_037554.1:20660384 (*GRM1*). Combining these findings with the critical reproductive efficiency metric, calving interval (unpublished data), the analysis reveals significant differences in calving interval across different genotypes at this locus.

For *GRM1* (NC_037554.1:20660384): The A/A and G/A genotype is associated with a significantly lower calving interval (year) compared to the G/G genotypes.

These findings highlight the influence of specific genotypes on Fertility, providing valuable insights for genetic selection and breeding programs aimed at improving fertility in water buffaloes.

## 4. Discussion

### 4.1. Population Stratification

Population stratification is a critical factor in GWAS studies, as it can introduce false associations due to differences in ancestral origins, leading to discrepancies in allele frequencies [29,30]. To mitigate this risk, we must account for population stratification in our dataset. Principal Component Analysis (PCA) helps reduce data complexity while preserving the covariance structure [31]. We used PLINK to calculate the proportion of variance explained by the first 10 principal components with filtered SNP markers, assessing how well these components capture genetic variation.

Additionally, we performed K-cluster analysis using Admixture software and evaluated cross-validation error (CV-error) to determine the optimal number of subpopulations. By minimizing CV-error, we identified the most appropriate value of K for population stratification. Despite being raised on the same farm, the Nili-Ravi buffaloes originate from Pakistan, while the Murrah buffaloes come from India. Our PCA plot shows clustering between groups MB and ZB, indicating a closer genetic relationship between these two groups compared to the others. Overall, the data suggest two main segments, but the CV-error plot indicates that the optimal number of subpopulations (K) is three. Therefore, we conclude that these 100 water buffaloes should be divided into three subgroups.

### 4.2. Genome-Wide Association Analysis of Reproductive-Related Traits

Enhancing the reproductive rate of water buffalo has become a critical research priority within the dairy industry. Water buffaloes are crucial for global dairy production, especially in regions where they are the main milk source. However, there is a significant research gap in understanding their fertility traits. Unlike Holstein cows, which have been extensively studied, water buffaloes have received much less attention. This disparity means that key aspects of water buffalo genetics, particularly those influencing reproductive performance, remain underexplored. Current breeding programs for water buffaloes lag behind those for other dairy species due to this lack of genetic insight. Identifying candidate genes and pathways that affect reproductive traits—such as calving interval, age at first calving, and conception rates—could greatly enhance breeding strategies. Improving these traits would increase the number of calves produced and boost overall herd productivity. Addressing this research gap is essential for developing targeted breeding strategies that focus on enhancing reproductive performance, thereby unlocking the full potential of water buffalo dairy operations.

In the association analysis of AFC, we identified a total of one candidate gene (*GRM1*). Fertility is a highly complex process involving the coordinated action of multiple signaling pathways. Among the identified genes, several are associated with key pathways, including the FOXO signaling pathway, calcium signaling pathway, and estrogen signaling pathway.

Previous studies have indicated that the FOXO signaling pathway plays a crucial role in regulating cellular growth, development, cell cycle progression, and other functions, all of which contribute to reproductive efficiency [32,33,34,35]. We have identified the candidate gene *GRM1* as playing a significant biological role within the FOXO signaling pathway. Specifically, *GRM1* (Metabotropic Glutamate Receptor 1) is involved in modulating various intracellular signaling cascades, including those related to oxidative stress response and apoptosis, both of which are critical for maintaining optimal reproductive performance [36,37]. For example, *GRM1* can interact with key components of the FOXO signaling pathway, such as *AKT* and *FOXO* transcription factors, to regulate cellular processes [38]. This interaction helps maintain cellular homeostasis and promotes efficient reproductive functions. By modulating the activity of *AKT*, *GRM1* can influence the phosphorylation status of *FOXO* proteins, thereby controlling their nuclear localization and transcriptional activity. This regulatory mechanism ensures that cells respond appropriately to environmental cues and maintain proper reproductive function.

### 4.3. The Mechanism of SNP Mutation and Fertility Traits

Among the selected SNPs, none were found to be non-synonymous. However, we identified base mutations in non-coding regions, specifically at position NC_037554.1:20660384 within the *GRM1* gene, which may potentially influence fertility-related traits. These findings suggest that variations in non-coding regions could play a crucial role in regulating important production and quality traits. Variability in non-transcriptional regulatory sequences, such as promoters, enhancers, and CTCF binding sites, is closely linked to the mechanisms underlying non-coding variation in cell development. However, whether these principles apply specifically to fertility traits requires further investigation [39,40].

### 4.4. Discussion of Tradeoffs

In dairy cows, extensive research has demonstrated that selection for increased milk production often comes at the expense of reproductive performance. This phenomenon can be attributed to several factors, including the following: Energy Allocation: High-producing cows allocate a significant portion of their energy resources to milk synthesis, leaving less available for other physiological functions, such as reproduction. This can result in delayed first calving (AFC), reduced conception rates, and prolonged intervals between calvings [41,42,43,44,45]. Nutritional Stress: Cows producing higher volumes of milk require more energy-dense diets to meet their nutritional needs. In environments where feed quality or availability is limited, breeding for lower milk production may be advantageous, as it allows for better allocation of energy to reproductive functions, thereby improving overall reproductive efficiency [46,47,48]. Physiological Strain: The metabolic demands of high milk production can induce stress on the cow’s body, potentially leading to health issues such as ketosis, mastitis, and lameness, all of which can negatively impact reproductive performance [49,50,51,52].

In areas where buffaloes are subjected to lower-quality feeds or seasonal variations in resource availability, breeding strategies should consider balancing milk production with reproductive efficiency. Selecting for moderate milk yields could enhance reproductive performance, ensuring sustainable herd management. Future studies should explore the genetic basis of these tradeoffs in buffaloes. Understanding the specific genes and pathways involved in both milk production and reproduction can help develop targeted breeding programs that optimize both traits. This makes it possible to develop integrated breeding programs that aim to balance milk production with reproductive performance, taking into account environmental constraints and feed availability.

### 4.5. Limitations and Future Directions

While our study provides valuable insights into the reproductive performance of buffaloes, particularly focusing on the age at first calving (AFC), there are several limitations that need to be acknowledged. Addressing these limitations will help guide future research and improve the robustness of our findings. Limitations: Sample Size: Our pilot study included a limited number of buffaloes, which may have affected the generalizability of our results. A larger sample size would allow for more comprehensive statistical analyses and potentially uncover additional factors influencing AFC and reproductive efficiency [3,53]; Parity (Litter Number): The study did not account for variations in parity, which could influence AFC and subsequent reproductive performance. Including data on multiple parities in future studies would provide a more nuanced understanding of how parity affects reproductive traits over time [54,55]; Calving Season: The timing of calving can significantly affect various reproductive parameters, including AFC. Our study did not control for or analyze the effects of different calving seasons. Future research should consider the seasonal variations in environmental conditions and management practices, as these factors can influence reproductive performance [56,57].

To build on this pilot study, we propose the following next steps: Expand the Sample Size: Conducting a larger-scale study with a broader geographic distribution and increased sample size will enhance the reliability and external validity of our findings [58]; Comprehensive Reproductive Performance Analysis: Future work should incorporate a holistic approach to reproductive performance by integrating data on AFC, first lactation performance, parity, and calving season. This integrated analysis will provide a more comprehensive understanding of the factors influencing reproductive efficiency; and Longitudinal Studies: Implement longitudinal studies to track changes in reproductive performance over multiple calving cycles and across different seasons. Such studies will offer deeper insights into the long-term impacts of AFC and other reproductive traits.

By addressing these limitations and pursuing these future directions, we aim to refine our understanding of the key determinants of reproductive performance in buffaloes and contribute to the development of more effective breeding and management strategies.

## 5. Conclusions

In this investigation, a genome-wide association analysis was conducted to identify genetic factors associated with reproduction-related traits in domestic water buffaloes, with a particular focus on age at first calving (AFC). Our study identified a total of two significant SNP loci, and one candidate gene within a 50 Kb range surrounding these loci was selected for further investigation. The candidate gene was enriched in biological processes such as FOXO signaling pathway (*GRM1*), calcium signaling pathway (*GRM1*), and estrogen signaling pathway (*GRM1*), all of which are directly or indirectly involved in the reproduction process. These findings offer a reference framework for comprehending the genetic architecture underlying production and quality attributes in water buffaloes, thereby paving the way for subsequent biological validation of the implicated genes. This is crucial guidance for breeding and improvement programs in water buffaloes. In future studies, we will focus on elucidating the relationships between candidate genes and metabolites involved in the production process. Additionally, longitudinal studies and functional validations are needed to confirm the roles of these genes in different environmental and management contexts. The aim is to uncover the metabolic pathways and mechanisms through which the genes influence production and composition.

## Figures and Tables

**Table 1:** Statistical description of fertility traits *.

Traits	Mean	SD	Min	Max
AFC	3.67	0.88	2.17	6.54

* SD, standard deviation. AFC, age at first calving.

**Table 2:** The SNPs identified via genome-wide association analysis encompass detailed information regarding their chromosomal locations, *p*-values, and associated candidate genes.

Methods	SNP	Chr	Pos	*p*	R2	Candidate Genes
SUPER/GLM(Q)/Blink/FarmCPU	1	NC_037554.1	20,660,384	5.0621 × 10^−8^	0.49381	*GRM1*
SUPER/GLM(Q)/Blink/FarmCPU	2	NC_037556.1	50,669,172	6.5734 × 10^−8^	0.42667	--
SUPER/GLM(Q)	3	NC_037545.1	35,877,289	6.8853 × 10^−8^	0.34081	--
SUPER/GLM(Q)	4	NC_037546.1	31,601,350	3.7412 × 10^−9^	0.41127	*OPN5*
SUPER/GLM(Q)	5	NC_037548.1	4,405,934	6.4571 × 10^−8^	0.33847	*FAM118A*; *UPK3A*; *KIAA0930*
SUPER/GLM(Q)	6	NC_037548.1	157,620,852	5.3121 × 10^−8^	0.3642	--
SUPER/GLM(Q)	7	NC_037551.1	20,700,724	8.906 × 10^−9^	0.3905	--
SUPER/GLM(Q)	8	NC_037551.1	52,947,579	1.05 × 10^−7^	0.35825	--
SUPER/GLM(Q)	9	NC_037552.1	24,460	6.7407 × 10^−8^	0.29687	--
SUPER/GLM(Q)	10	NC_037552.1	112,491,377	3.2491 × 10^−8^	0.32257	*ZNF777*; *ZNF746*
SUPER/GLM(Q)	11	NC_037552.1	113,607,118	2.4523 × 10^−8^	0.33352	*ABCB8*; *ASIC3*; *CDK5*; *SLC4A2*; *FASTK*; *TMUB1*; *AGAP3*
SUPER/GLM(Q)	12	NC_037553.1	15,992,633	2.4361 × 10^−8^	0.36112	*FAM81B*
SUPER/GLM(Q)	13	NC_037554.1	9,287,335	9.7346 × 10^−8^	0.31436	*TULP4*; *GTF2H5*; *SERAC1*
SUPER/GLM(Q)	14	NC_037554.1	16,312,432	1.3425 × 10^−9^	0.3879	--
SUPER/GLM(Q)	15	NC_037554.1	99,978,913	1.0129 × 10^−7^	0.33706	--
SUPER/GLM(Q)	16	NC_037555.1	32,169,042	1.5181 × 10^−7^	0.33459	--
SUPER/GLM(Q)	17	NC_037556.1	75,679,149	8.5899 × 10^−8^	0.36278	--
SUPER/GLM(Q)	18	NC_037556.1	80,488,833	1.413 × 10^−8^	0.39141	*KCNS3*; *MSGN1*
SUPER/GLM(Q)	19	NC_037556.1	81,118,534	7.4243 × 10^−9^	0.36956	--
SUPER/GLM(Q)	20	NC_037558.1	8,094,566	8.613 × 10^−8^	0.37056	*YBX1*; *SLC2A10*
SUPER/GLM(Q)	21	NC_037559.1	57,471,045	5.8845 × 10^−8^	0.32884	--
SUPER/GLM(Q)	22	NC_037559.1	74,176,040	3.5625 × 10^−8^	0.35173	*WISP1*; *NDRG1*
SUPER/GLM(Q)	23	NC_037561.1	25,915,631	2.8368 × 10^−9^	0.40653	--
SUPER/GLM(Q)	24	NC_037561.1	25,915,784	1.0496 × 10^−9^	0.40948	--
SUPER/GLM(Q)	25	NC_037562.1	28,221,520	6.038 × 10^−8^	0.34565	--
SUPER/GLM(Q)	26	NC_037562.1	28,221,526	6.038 × 10^−8^	0.34565	--
SUPER/GLM(Q)	27	NC_037562.1	28,221,527	6.038 × 10^−8^	0.34565	--
SUPER/GLM(Q)	28	NC_037562.1	39,920,231	2.277 × 10^−9^	0.40894	*HYDIN*
SUPER/GLM(Q)	29	NC_037563.1	45,012,914	1.8896 × 10^−8^	0.36036	--
SUPER/GLM(Q)	30	NC_037564.1	36,725,181	9.3193 × 10^−8^	0.31929	*LINGO1*
SUPER/GLM(Q)	31	NC_037566.1	36,784,161	4.9686 × 10^−8^	0.3255	*B4GALT6*
SUPER/GLM(Q)	32	NC_037568.1	13,951,865	6.9031 × 10^−8^	0.33258	*CALN1*
SUPER/GLM(Q)	33	NC_037569.1	5,832,499	2.107 × 10^−8^	0.35009	*KAL1*
SUPER/GLM(Q)	34	NC_037569.1	38,961,581	1.0446 × 10^−7^	0.29241	*BCOR*

**Table 3:** The results of individual genotyping.

Candidate Gene	SNP (Chr:Pos)	Calving Interval (Year)		
		Homozygous Mutation	Heterozygous Mutation	Reference Genotype
*GRM1*	NC_037554.1:20660384	A/A	G/A	G/G
		1.02 ± 0.15 B	1.21 ± 0.23 B	1.52 ± 0.28 A

The phenotypic values of calving interval (year) are expressed as “least squares mean ± standard deviation”. Different letters in the same column indicate significant differences (*p* < 0.05); the same letter or no letter indicates no significant difference (*p* > 0.05). The genotypes of SNP loci are arranged in the order of homozygous mutant, heterozygous, and reference types.

## Data Availability

Restrictions apply to the availability of these data. Data were obtained from EMBL-EBO and are available at https://www.ebi.ac.uk/eva/?eva-study=PRJEB72029 (accessed on 19 May 2024) with the permission of EMBL-EBO.

## Supplementary Materials

The following supporting information can be downloaded at: https://www.mdpi.com/article/10.3390/genes16020193/s1, Supplementary Table S1: Detailed pathway information for these candidate genes. Supplementary Table S2: KEGG pathways of candidate genes.
